# Dietary modification reduces serum angiopoietin-like protein 2 levels and arterial stiffness in overweight and obese men 

**DOI:** 10.20463/jenb.2019.0021

**Published:** 2019-09-30

**Authors:** Jiyeon Park, Youngju Choi, Ryoko Mizushima, Toru Yoshikawa, Kanae Myoenzono, Kaname Tagawa, Masahiro Matsui, Kiyoji Tanaka, Seiji Maeda

**Affiliations:** 1. Graduate School of Comprehensive Human Sciences, University of Tsukuba, Ibaraki Japan; 2. Faculty of Health and Sport Sciences, University of Tsukuba, Ibaraki Japan; 3. Department of Nutritional Epidemiology and Shokuiku, National Institute of Health and Nutrition, National Institutes of Biomedical Innovation, Health and Nutrition, Tokyo Japan; 4. Faculty of Health and Sport Sciences, Ryutsu Keizai University, Ibaraki Japan; 5. Graduate School of Education, Miyagi University of Education, Miyagi Japan

**Keywords:** ANGPTL2, arterial stiffness, dietary modification, obesity

## Abstract

**[Purpose]:**

Weight loss can reduce obesity-induced arterial stiffening that is attributed to decreased inflammation. Angiopoietin-like protein 2 (ANGPTL2) is a pro-inflammatory adipokine that is upregulated in obesity and is important in the progression of atherosclerosis and cardiovascular disease. The purpose of this study is to investigate the effects of dietary modification on circulating ANGPTL2 levels and arterial stiffness in overweight and obese men.

**[Methods]:**

Twenty-two overweight and obese men (with mean age of 56 ± 2 years and body mass index of 28.6 ± 2.6 kg/m^2^) completed a 12-week dietary modification program. We measured the arterial compliance and β-stiffness index (as the indices of arterial stiffness) and serum ANGPTL2 levels before and after the program.

**[Results]:**

After the 12-week dietary modification, body mass and daily energy intake were significantly reduced. Arterial compliance was significantly increased and β-stiffness index was significantly decreased after the 12-week dietary modification program. Serum ANGPTL2 levels were significantly decreased. Also, the changes in arterial compliance were negatively correlated with the changes in serum ANGPTL2 levels, whereas the changes in β-stiffness index were positively correlated with the changes in serum ANGPTL2 levels.

**[Conclusion]:**

These results suggest that the decrease in circulating ANGPTL2 levels can be attributed to the dietary modification-induced reduction of arterial stiffness in overweight and obese men.

## INTRODUCTION

Obesity has become a major health concern globally and is closely linked to increasing arterial stiffness^1^^,^^2^ that is a primary risk factor for cardiovascular disease (CVD). Increasing evidence indicates that obesity is linked to chronic and systemic inflammation^3^^,^^4^. Chronic low-grade inflammation along with an excess of adipose tissue releases a variety of cytokines and bioactive mediators that is important in several cardiovascular disorders. Arterial stiffness is associated with the production of pro-inflammatory adipokines in obese individuals^5^^,^^6^. It has been demonstrated that obese individuals are likely to exhibit increased arterial stiffness and pro-inflammatory biomolecules^2^^,^^3^^,^^5^.

Angiopoietin-like protein 2 (ANGPTL2) belongs to the angiopoietin-like family of proteins and is abundantly expressed in the adipose tissue, endothelial cells, and macrophages infiltrating atheromatous plaques^7^^-^^9^. ANGPTL2 in adipose tissue is higher in diet-induced obese mice than in control mice^7^ and circulating ANGPTL2 levels in obese individuals positively correlate with adiposity^10^, indicating that ANGPTL2 is a pro-inflammatory adipokine. Moreover, ANGPTL2 from perivascular adipose tissue and endothelial cell promotes local and vascular inflammation^8^^,^^11^. Recently, Hata et al. reported that ANGPTL2 levels are a novel marker for CVD in the general population^12^. Furthermore, elevated circulating ANGPTL2 is independently associated with carotid intima-media thickness (IMT) that is a predictor of CVD events^13^ in patients with type 2 diabetes mellitus^14^. Since pro-inflammatory adipokines contribute to arterial stiffening^15^, ANGPTL2 levels may be relevant for vascular function.

It has been reported that weight loss is effective in reducing arterial stiffness and levels of various pro-inflammatory biomolecules in obese individuals^5^^,^^16^^-^^18^. However, the association between weight loss-induced changes in arterial stiffness and circulating ANGPTL2 levels in the obese population remains unclear. The purpose of this study was to investigate the effects of dietary modification on circulating ANGPTL2 levels and arterial stiffness in overweight and obese men. We hypothesized that dietary modification decreases circulating ANGPTL2 levels which in turn reduces arterial stiffness in overweight and obese men.

## METHODS

### Participants

Individuals were invited to participate through an advertisement in the local newspaper. In accordance with the guidelines set by the World Health Organization, volunteers with a body mass index (BMI) of ≥25 kg/m^2^ were considered overweight and obese. A total of 22 overweight and obese men (with a mean age and BMI of 56 ± 2 years and 28.6 ± 2.6 kg/m^2^, respectively) completed a 12-week dietary modification program. None of the participants had cardiovascular or cerebrovascular disease, as determined by their medical history and physical examination. The study subjects included 5 current smokers and 10 participants on antihypertensive, antidiabetic, or antihyperlipidemic medication. This study was a prespecified sub-study (secondary analysis) that was registered under the UMIN (University Hospital Medical Information Network) clinical trials registry (UMIN000028214) and was approved by the Institutional Review Board of the University of Tsukuba (IRB approval number: Tai 28-143). All the experiments were carried out in accordance with The Code of Ethics of the World Medical Association (Declaration of Helsinki), and all the volunteers provided their written informed consent before participating.

### Study design

Anthropometric measurements, blood pressure and heart rate, carotid arterial compliance, and blood chemistry were determined in all the participants before and after the 12-week dietary modification program. Dietary intake was estimated using a questionnaire before and after the program. Participants abstained from caffeine and alcohol and fasted for at least 12 hours before being evaluated. Immediately prior to assessment, participants rested in the supine position for a minimum of 20 minutes in a quiet and temperature-controlled room (25°C).

### Dietary modification

The dietary modification program consisted of weekly instructional group sessions and individual counseling sessions conducted by trained staff for 12 weeks (2 hours per session and 8 sessions in total). The participants were instructed to consume nutritionally well-balanced meals (comprising proteins, carbohydrates, fats, amino acids, vitamins, and minerals) similar to previous studies^16^^,^^17^^,^^19^. The dietary modification program included four food groups^20^^,^^21^ and aimed at limiting the total daily energy intake to 1,680 kcal (21 points; 1 point=80 kcal) and allowed a self-selection of foods in the groups. Daily energy intake limits were as follows: 240 kcal (3 points) from food group I (e.g., dairy products and eggs), 240 kcal (3 points) from food group II (e.g., meat, fish, and beans), 240 kcal (3 points) from food group III (e.g., vegetables, fruits, and seaweeds), and 960 kcal (12 points) from food group IV (e.g., grains, nuts, snacks, oil, sugar, beverages, and alcohol). During the program, the participants were asked to record their daily food consumption in a diary in which trained dietitians added their compliments and advice during the weekly group sessions. The participants were individually counseled and encouraged directly after the instructional sessions.

### Anthropometric measurements

Anthropometric measurements were performed on barefoot and lightly clothes participants. Body mass was measured to the nearest 0.1 kg using a calibrated digital scale (InBody 770; InBody Japan, Tokyo, Japan). Height was measured to the nearest 0.1 cm using a wall-mounted stadiometer (Digital Height Meter AD-6227 by A&D, Tokyo, Japan). BMI values were calculated by dividing the weight (kg) by the square of the height (m). Total body fat was estimated using bioelectrical impedance analysis (InBody 770). Waist circumference was measured on the skin at the umbilicus in the standing position. Abdominal visceral fat area was determined using the dual-impedance analysis method (HDS-2000; Omron Healthcare, Kyoto, Japan) that is efficient in calculating simple abdominal visceral fat accumulation. The measurements were performed in the supine position. A strong correlation was reported between the abdominal visceral fat area determined by the HDS-2000 and abdominal visceral fat area determined by a CT scan (*r*=0.888, *p*<0.001)^22^. Waist circumference and abdominal visceral fat area were measured in duplicates to the nearest 0.1 cm and 1 cm^2^, respectively.

### Blood pressure and heart rate

Systolic blood pressure (SBP), diastolic blood pressure (DBP), and heart rate (HR) of the volunteers were measured by the oscillometric method using a non-invasive and semi-automated vascular profiling system (form PWV/ABI, Colin Medical Technology, Komaki, Japan) in the supine position.

### Arterial compliance and β-stiffness index

Arterial compliance was determined by a combination of ultrasound imaging of a pulsatile carotid artery and simultaneous applanation of tonometrically obtained arterial pressure using the contralateral carotid artery^23^. Diameter of the right common carotid artery was measured from the images obtained by an ultrasound device (En Visor; Koninklijke Philips Electronics, Eindhoven, the Netherlands) equipped with a high-resolution linear array transducer. A longitudinal image of the cephalic portion of the common carotid artery was acquired 1–2 cm proximal to the carotid bulb. All image analyses were performed by one investigator using ImageJ (National Institutes of Health, Bethesda, MD, USA). The minimum and maximum arterial lumen diameters were measured at 3 points in each frame and the mean values were calculated. The pressure wave forms of the left common carotid artery were recorded with an applanation tonometry device (form PWV/ABI) and calibrated by equating the carotid mean arterial and diastolic blood pressure to that of the brachial artery. Arterial compliance was calculated using the following equation: [(D1 − D0) / D0] / [2(P1 − P0)] π(D0)^2^. The β-stiffness index, a parameter for determining central arterial stiffness independent from blood pressure^24^, was calculated using the following equation: ln (P1 / P0) / [D1 − D0] / D0]. In each equation, “P” and “D” represented the pressure and diameter, respectively; and, “1” and “0” referred to the maximum (systolic) and minimum (diastolic) values for pressure and diameter during the cardiac cycle^25^. 

### Serum ANGPTL2 levels and blood chemistry

Blood samples were collected from each participant in the morning after a 12-hour overnight fast. Each blood sample was placed in a serum separator tube and centrifuged at 2,000 rpm for 15 minutes at 4°C. The serum were stored at -80°C until further analysis. Serum ANGPTL2 levels were measured by ELISA using the Human ANGPTL2 Assay (Code No. 27745, Immuno-Biological Laboratories, Tokyo, Japan), according to the kit protocol. Serum levels of triglycerides, total cholesterol, high-density lipoprotein cholesterol (HDL), and low-density lipoprotein cholesterol (LDL) were determined using standard enzymatic techniques. 

### Survey of dietary intake 

Daily dietary intake was recorded using a food frequency questionnaire (FFQg) that included 29 food groups and 10 cooking methods. This method can be used for a variety of clinical investigations^26^. The questionnaire assessed the average daily intake frequency and total intake per week for each food or food group in commonly used units or portion sizes^27^. The nutrient contents were analyzed using the Excel Eiyo-kun FFQg version 4.0 software (Kenpaku-sha, Tokyo, Japan).

### Data analysis

Values were represented as mean ± standard error. The normality of all parameters was assessed by the Shapiro-Wilk test. The paired Student’s t-test and Wilcoxon signed-rank test were used to analyze the variables before and after the dietary program. The correlation between changes in arterial compliance and changes in serum ANGPTL2 levels, and changes in β-stiffness index and changes in serum ANGPTL2 levels after the program were determined using the Pearson’s correlation coefficient. The partial correlation coefficient was adjusted for age or age and medication. We performed a stepwise multiple regression analysis to determine the effects of the changes in BMI, visceral adipose area, SBP, and ANGPTL2 levels on arterial compliance. All statistical analyses were performed using SPSS 23.0 (IBM, Armonk, NY, USA) considering statistical significance at *p*<0.05.

## RESULTS

**Figure 1.**
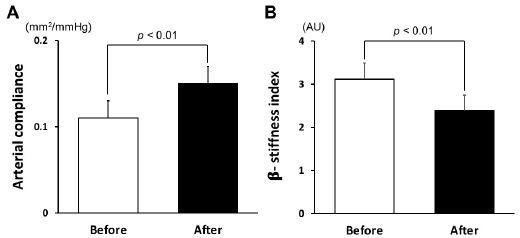
Arterial compliance (A) and β-stiffness index (B) before and after the 12-week dietary modification program.

**Figure 2.**
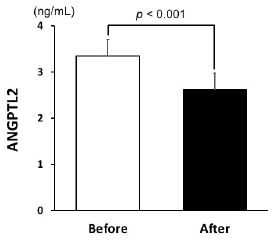
Serum ANGPTL2 levels before and after the 12-week dietary modification program.

**Figure 3.**
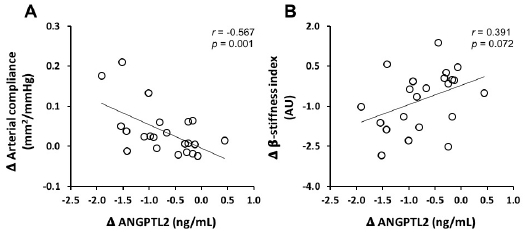
Correlation between the changes in ANGPTL2 and arterial compliance (A) and β-stiffness index (B) following the 12-week dietary modification program.

Table 1 shows the characteristics of the participants before and after the 12-week dietary modification program. Body mass, BMI, total body fat, skeletal muscle mass, waist circumference, abdominal visceral fat area, total cholesterol, LDL cholesterol, triglycerides, blood pressure, and heart rate of the participants significantly decreased after the 12-week dietary modification program. Energy intake and major nutrients (i.e., carbohydrates, proteins, and fats) also decreased significantly after the program. Arterial compliance increased significantly, while β-stiffness indices significantly decreased after this program (Figure 1). Serum ANGPTL2 levels reduced after the 12-week dietary modification program (Figure 2). We found significant correlation between changes in serum ANGPTL2 levels and changes in arterial compliance regardless of whether the comparison was unadjusted (*r*=-0.57, *p*<0.01), adjusted for age and medication (*r*=-0.64, *p*<0.01), or adjusted for age, medication, and smoking habits (*r*=-0.66, *p*<0.01). Moreover, the changes in serum ANGPTL2 levels were associated with changes in β-stiffness index (*r*=0.39, *p*=0.07) and showed significant correlation when adjusted for age and medication (*r*=-0.55, *p*<0.05) and age, medication, and smoking habits (*r*=0.60, *p*<0.01; Figure 3). Stepwise multiple regression analysis revealed that changes in arterial compliance were significantly associated with changes in serum ANGPTL2 levels (β=-0.41, *p*<0.05) and abdominal visceral fat area (β=-0.47, *p*<0.05) area, whereas changes in BMI (β=0.24, *p*=0.33) and SBP (β=-0.10, *p*=0.65) were not associated with changes in arterial compliance.

**Table 1.** Characteristics of the participants before and after the 12-week dietary modification

	Before	After
Age, years	56 ± 2	
Height, cm	170.1 ± 1.4	
Body mass, kg	82.6 ± 2.6	77.0 ± 2.6***
Body mass index, kg/m^2^	28.6 ± 0.7	26.7 ± 0.8***
Total body fat, %	29.8 ± 1.1	27.0 ± 1.3***
Skeletal muscle mass, kg	32.4 ± 1.0	31.2 ± 0.9***
Waist circumference, cm	99.6 ± 1.7	92.8 ± 1.7***
Abdominal visceral fat area, cm^2^	126.9 ± 9.5	91.9 ± 8.5***
Total cholesterol, mg/dL	195 ± 6	183 ± 6*
HDL cholesterol, mg/dL	50 ± 2	48 ± 2
LDL cholesterol, mg/dL	124 ± 5	113 ± 95**
Triglycerides, mg/dL	126 ± 10	101 ± 8***
Systolic blood pressure, mm Hg	127 ± 3	123 ± 3*
Diastolic blood pressure, mm Hg	80 ± 2	77 ± 2**
Heart rate, beats/min	59 ± 2	56 ± 1*
Total Energy intake, kcal/day	2643 ± 191	1562 ± 83***
Carbohydrate intake, g/day	368 ± 20	198 ± 10***
Protein intake, g/day	84 ± 6	61 ± 4**
Fat intake, g/day	75 ± 6	53 ± 4**

Values are means ± SE. Abbreviations: HDL, high-density lipoprotein; LDL, low-density lipoprotein.

*p < 0.05, **p < 0.01, ***p < 0.001 vs. before the intervention

## DISCUSSION

In this study, we investigated how 12 weeks of dietary modification affects circulating ANGPTL2 levels and arterial stiffness in overweight and obese men. We observed that dietary modification reduced both circulating ANGPTL2 levels and arterial stiffness. Moreover, we found that the dietary modification-induced reduction in circulating ANGPTL2 levels correlated with changes in arterial compliance and β-stiffness index. Multiple regression analysis revealed that an increase in arterial compliance was accompanied with a decrease in ANGPTL2 levels and abdominal visceral fat area in overweight and obese men. These results suggest that dietary modification in overweight and obese men reduces circulating ANGPTL2 levels that contributes to the decreases of arterial stiffness.

Metabolic homeostasis is achieved by balanced expression of pro- and anti-inflammatory adipokines in healthy individuals^3^. However, in obesity, adipose tissue dysfunction leads to the upregulation of pro-inflammatory adipokines and downregulation of anti-inflammatory adipokines, resulting in a chronic and systemic inflammatory state. Circulating ANGPTL2, a pro-inflammatory adipokine, is abundantly expressed in white adipose tissue in mice, especially in the visceral adipose tissue^8^. ANGPTL2 overexpression in adipose tissues leads to an increase in adipose tissue inflammation^3^, suggesting that adipose tissue is the best source of circulating ANGPTL2. It has been reported that weight loss after lifestyle modification counseling significantly reduces ANGPTL2 levels in obese men^10^. However, lifestyle modification in this study included only one motivational lecture on weight loss and support program (i.e., behavioral target setting and e-mail support). In our study, we selected a dietary modification method for the weight loss of obese individuals with well-balanced dietary restrictions17. To the best of our knowledge, this is the first study to report a significant reduction in circulating ANGPTL2 levels after dietary modification for weight loss in overweight and obese men, suggesting that better protocols can be devised to elucidate the effect of dietary modification on ANGPTL2. 

There is strong evidence that increasing levels of pro-inflammatory markers have a major role in the development of arterial stiffness^2^^,^^28^. It is known that ANGPTL2 is an important player in local and vascular inflammation^3^. Circulating ANGPTL2 levels were found to positively correlate with carotid IMT in patients with type 2 diabetes mellitus^14^ and carotid-femoral pulse wave velocity in patients with chronic kidney disease^29^. Long-term follow-up study has shown that higher serum ANGPTL2 levels increase the frequency of CVD, including coronary heart disease and stroke in the Japanese population^12^. Therefore, based on earlier studies, increased ANGPTL2 may trigger arterial stiffness. In this study, we have shown the reduction in both circulating ANGPTL2 levels and arterial stiffness in overweight and obese men after dietary modification. Moreover, we found that dietary modification-induced changes in ANGPTL2 significantly correlate with changes in arterial stiffness. Multiple regression analysis revealed that changes in serum ANGPTL2 levels and abdominal visceral fat area independently affected the changes in BMI and blood pressure. These findings indicated that a reduction in ANGPTL2 upon weight loss improves the flexibility of arteries.

In this study, we observed that dietary modification decreases circulating ANGPTL2 levels in overweight and obese men. However, the source of the dietary modification-induced decrease in circulating ANGPTL2 levels remains unclear. Arterial stiffness is influenced by endothelial function and progression of atherosclerosis^26^. ANGPTL2 is expressed in endothelial cells as well as in adipose tissue. It has been previously reported that ANGPTL2 expression is observed in atheromatous plaques in patients with coronary heart disease, particularly in endothelial cells and infiltrated macrophages^8^. This suggests that ANGPTL2 from these cells may contribute to endothelial dysfunction and progression of atherosclerosis. Meta-analysis reviews have reported that long-term healthy dietary modification may potentially modulate endothelial function by increasing the bioavailability of vasoactive mediators and reducing oxidative stress and pro-inflammatory mediators^5^^,^^18^. Therefore, it is possible that a reduction in ANGPTL2 levels after long-term dietary modification ameliorates endothelial function, which is related to arterial stiffness, in overweight and obese men. Further research is warranted to efficiently understand these correlations and mechanisms responsible for the observed changes in overweight and obese individuals. 

However, there are several limitations in this study, such as a limited number of participants and no comparable control groups. Moreover, it has been reported that intake of omega-3, soy isoflavones, salt, fermented milk products, tea, and caffeine have favorable effects on arterial stiffness and endothelial function^18^. However, we could not determine the dietary composition even though all the participants were assessed for their daily intake frequency and total intake using the food questionnaires. Thus, randomized controlled trials with larger sample sizes or a variety of methods are needed in the future to determine the effects of dietary modification on ANGPTL2 levels and arterial stiffness in overweight and obese individuals. 

In conclusion, our study showed that dietary modification for 12 weeks reduces circulating ANGPTL2 levels and arterial stiffness in overweight and obese men. To the best of our knowledge, this is the first study to investigate a relationship between dietary modification-induced changes in circulating ANGPTL2 levels and arterial stiffness. This indicates that dietary modification-induced reduction in ANGPTL2 leads to reduced arterial stiffness in overweight and obese men.
